# Deep Eutectic Solvent Interaction with Graphene Oxide: A Combined Experimental and Molecular Dynamics Characterization

**DOI:** 10.1021/acs.jpcb.5c03461

**Published:** 2025-08-26

**Authors:** Simone Di Muzio, Fabio Ramondo, Giulia Fioravanti

**Affiliations:** † Istituto di Fotonica e Nanotecnologie, Consiglio Nazionale delle Ricerche, P.zza Leonardo da Vinci 32, Milan 20133, Italy; ‡ Istituto dei Sistemi Complessi, Consiglio Nazionale delle Ricerche, P.le Aldo Moro 7, Rome 00185, Italy; § Department of Chemistry, Sapienza Università di Roma, P.le Aldo Moro 5, Rome 00185, Italy; ∥ Department of Physical and Chemical Sciences, University of L’Aquila, Via Vetoio, L’Aquila 67100, Italy

## Abstract

The interaction between graphene oxide (GO) and deep eutectic solvents (DESs) plays a crucial role in the design of functional materials for a wide range of applications. In this study, we present a combined experimental and computational investigation aimed at elucidating the structural and molecular organization of GO–DES systems using ethaline and reline as model deep eutectic solvents. These two DESs are among the most widely studied and well-characterized, making them ideal benchmarks for probing GO–liquid interactions. We synthesized GO and performed a detailed characterization via X-ray photoelectron spectroscopy (XPS), obtaining precise information about the type and distribution of oxygen-containing functional groups. Based on these experimental data, we developed a realistic molecular model of GO, providing a reliable and reproducible framework for atomistic simulations. Infrared and Raman spectroscopies reveal specific changes in vibrational modes upon GO–DES interaction, while differential scanning calorimetry (DSC) indicates modifications in thermal behavior. Classical molecular dynamics (MD) simulations show the formation of hydrogen-bond networks between the DES components and GO surface functionalities. Our results demonstrate a reciprocal structural influence between GO and DES at the molecular level and establish a validated computational protocol for the study of these hybrid systems.

## Graphical abstract

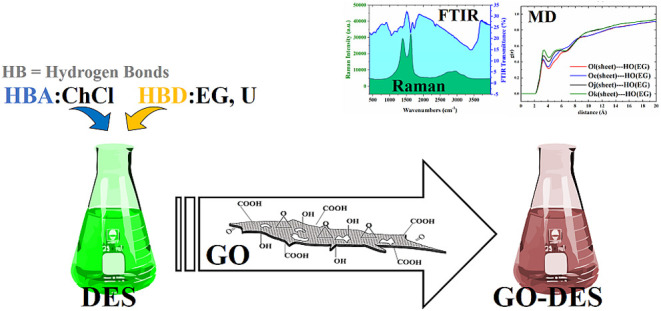

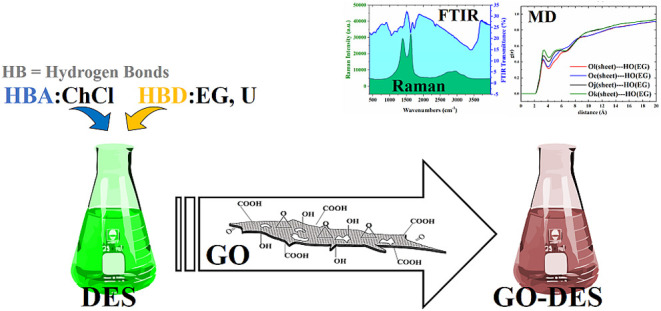

## Introduction

Graphene oxide (GO) has emerged as a remarkable material with a wide range of potential applications due to its unique properties, including high surface area, excellent mechanical strength, and tunable electronic properties. Derived from graphite through oxidative processes,^1^ GO is rich in oxygen-containing groups, which impart hydrophilicity and facilitate functionalization, making it a versatile platform for numerous applications, including energy storage,^2,3^ sensors,^4–6^ and biomedical devices.^7,8^ In recent years, the integration of GO with deep eutectic solvents (DES) has garnered significant attention from researchers and scientists, as the combination of these two materials has the potential to unlock new and innovative applications in various fields.^9–13^ Deep eutectic solvents (DES) are a new class of sustainable and environmentally friendly solvents composed of two or more components that form a liquid with a melting point significantly lower than that of either individual component.^14^ DESs are characterized by their low toxicity,^15,16^ biodegradability,^17^ and ease of preparation. They possess unique physicochemical properties, such as low volatility, high thermal stability, and tunable solubility, which have garnered interest for applications in green chemistry, electrochemistry, and materials science.^18–20^

GO–DES composites leverage the synergistic effects of both constituents, combining the structural and functional versatility of GO with the favorable solvent properties of DESs. The result is a material with enhanced properties that can be fine-tuned for advanced applications. The use of GO–DES composites in Solid Phase Extraction (SPE) has garnered significant attention due to the enhanced adsorption capacity, selectivity, and environmental sustainability of the composite.^21–24^ The combination of GO with DESs can significantly improve the electrochemical performance. For instance, P-doped GO electrodes synthesized using ethaline, a mixture of choline chloride (ChCl) and ethylene glycol (EG) in a 1:2 molar ratio,^25^ exhibited high capacitance and outstanding cycling stability, making them effective materials for supercapacitors.^26^ Reline, obtained by combining ChCl and urea in a 1:2 molar ratio,^27^ has been explored as a green, nonflammable electrolyte for carbon-based supercapacitors, offering a broad voltage window and extended lifespan. In the realm of energy storage, these composites can improve the performance of supercapacitors and batteries due to their high ionic conductivity and structural integrity.^28–30^ In catalysis, the unique environment provided by DESs can enhance the catalytic activity and stability of GO-based catalysts.^31–33^ Furthermore, in biomedical applications, the biocompatibility and low toxicity of DESs, coupled with the functionalizable surface of GO, open new avenues for drug delivery systems and biosensors.^34^

Understanding the interactions between GO and DES represents a significant advancement in materials science, particularly in the context of green chemistry and sustainable materials, and is crucial for leveraging their combined properties in applications such as energy storage, catalysis, and environmental remediation. For instance, the adsorption of DES molecules on GO can modify its surface chemistry, affecting charge transfer and ion diffusion, which are vital for supercapacitor performance. In this study, we concentrated on examining the interactions of GO with two widely used DES: ethaline and reline. The intricate interplay between the surface functional groups of GO and the hydrogen-bonding network of DES profoundly affects their physicochemical properties. Specifically, such interactions affect the solubility and dispersion stability of GO, enhance its chemical modification potential, and simultaneously alter the structural organization and properties of DES. To comprehensively study these interactions, this work combines experimental and computational approaches. Spectroscopic techniques such as Infrared (FT-IR) and Raman spectroscopy (RS) offer experimental evidence of chemical bonding and vibrational changes in GO and DES upon interaction. Additionally, differential scanning calorimetry (DSC) is used to investigate the thermal behavior and phase transitions of the GO–DES mixtures, providing a holistic understanding of the interaction mechanisms. This information is complemented by Molecular Dynamics (MD) simulations, which provide atomistic insights into the binding mechanisms, hydrogen-bonding interactions, and structural organization of GO–DES systems, revealing the strong adsorption of DES molecules on graphene surfaces.

This integrated approach not only contributes to a deeper understanding of the fundamental interactions between GO and DES but also paves the way for the development of advanced materials with tailored properties for specific applications. By coupling molecular dynamics with advanced characterization techniques, this study bridges computational predictions with experimental validation, shedding light on the dynamic and structural properties of graphene oxide in deep eutectic solvents.

## Experimental Methods

The synthesis procedures for GO, DES, and GO–DES composites are detailed in the Supporting Information file. Comprehensive characterization data, including X-ray Photoelectron Spectroscopy (XPS) and FTIR spectra of GO, as well as Raman spectra of the DESs and their individual components, are also thoroughly reported in the Supporting Information.

### Differential Scanning Calorimetry (DSC)

DSC measurements were carried out using a Mettler Toledo DSC 3 calorimeter (Mettler Toledo International Inc., Columbus, OH). GO and GO–DES composites were directly inserted into Aluminum pans of 40 μL. A single heating scan was performed from −80 up to 350 °C, at a heating rate of 10 °C/min. Low temperature cycling was performed from room temperature (RT) to −80 °C at 2 °C/min, isotherm step at 80 °C for 5 min, and heating segment from −80 °C up to RT at 2 °C/min. Under the experimental conditions, reproducible thermal recordings were obtained. The uncertainty on temperatures was ±0.1 °C and that on Δ*H* was ± 0.5 kJ/mol.

### Fourier-Transform Infrared Spectroscopy (FTIR)

FTIR spectra were performed on a PerkinElmer spectrophotometer Spectrum Two, equipped with a reflectance module (ATR). Measurements were carried out at room temperature over the spectral range of 4000–400 cm^–1^, using a resolution of 2 cm^–1^ and averaging 16 accumulated scans to ensure optimal signal quality and reproducibility. The samples were predried in an oven at a maximum temperature of 60 °C for a minimum of 24 h to ensure the removal of residual moisture before analysis.

### Raman Spectroscopy (RS)

RS was performed using a LABRAM system (Horiba-Jobin Yvon, Japan, λ = 633 nm, 1 μm spatial resolution, and about 2 cm^–1^ spectral resolution). The GO was measured directly on a dried sample, while the GO–DES samples were drop-casted onto a glass substrate.

### Computational Details

The GO sheet was generated by means of the GOPY script,^35^ a Python open-source tool developed for automatic generation of 2D materials. The side of the sheet was imposed to 80 × 80­(Å). In order to better describe the GO structure, according to our previous structural characterization,^36,37^ 13 holes were included on the sheet surface. Based on the XPS analysis results, the oxidation percentage was accounted for by putting 181 carbonyls (C=O), 417 epoxide (C–O–C), and 419 hydroxyl (C–OH) groups. The interactions of both DESs (reline and ethaline) with the GO surface were studied through classical molecular dynamics simulations by creating two cubic boxes with an initial side of 120 Å, each containing 2% of GO. According to the previous literature,^38–41^ the molar ratio between DES components was chosen as 1:2 (one part of choline chloride and two parts of urea or ethylene glycol). Each box contained a single graphene oxide sheet placed at the center of the simulation box, without applying any positional constraints, in order to preserve the physical realism of the simulation. The systems included 2500 choline chloride molecules and 5000 molecules of urea (for reline) or ethylene glycol (for ethaline). All molecular species were parametrized with OPLS-AA force fields,^42^ which have been shown to be highly effective for the computational study of both molecular^43,44^ and ionic liquids.^42,45,46^ Partial atomic charges were calculated at the HF/6–31G** level of theory by means of the Gaussian16 ^47^ package using the RESP algorithm:^48^ aiming to reduce the computational costs, small representative models of graphene substituted with functional groups such as C–OH, C–O–C, and C=O were used to calculate partial atomic charges. The simulations were carried out by means of the Gromacs2019.6 package^49^ under periodic boundary conditions. The electrostatic interactions were considered by Particle mesh Ewald (PME). All bonds containing a hydrogen atom were constrained with the LINCS algorithm^50^ in order to avoid any resonance effects. The radii of van der Waals interactions were truncated, imposing a cutoff of 10 Å. The simulation protocol consisted of an initial energy minimization (104 cycles), followed by a 1 ns simulation in the NVE ensemble. This brief microcanonical cycle was employed immediately after minimization to stabilize the system without introducing external constraints on temperature or pressure, allowing for a natural initial relaxation.^51–53^ Subsequently, the system was equilibrated for 2 ns in the NVT ensemble at 550 K. The use of high temperatures in this stage was intended to enhance the fluidity of the deep eutectic solvents and promote molecular and ionic mobility. The system was then cooled to 400 K and subjected to 20 ns of NPT simulation, followed by a final 30 ns of NVT production run. Temperature and pressure were controlled using the V-rescale thermostat and the Parrinello–Rahman barostat.^54,55^ The final trajectories were analyzed by Travis software.^56^ All simulations were carried out with a time step of 1 fs.

## Results and Discussion

### GO: Chemical–Physical Characterization

Accurate structural models of GO are critical for reliable molecular dynamics (MD) simulations, particularly when studying interactions with deep eutectic solvents, impacting not only solubility and stability but also the potential applications of these composites. Rigorous chemical–physical characterization ensures that simulations capture GO’s complex structure–property relationships, which directly influence its behavior in solvent environments. This work aims to highlight the role of multitechnique characterization in modeling GO by providing a dimensional model that, to the best of our knowledge, is not currently available in the literature. Considering that GO is, in fact, a class of materials rather than a single material, its structure is strongly dependent on the synthetic route followed.

The most widely adopted method for GO large-scale production involves the oxidation of graphite using concentrated acids in the presence of strong oxidant systems, followed by an exfoliation step. Over time, several synthetic methods have been used, mainly variants of methods based on different oxidant systems. The different methods can be classified into the Hummers (HU), Hofmann (HO), and Staudenmaier (ST) methods. In this work, we employed a modified Hummers’ method, which has been shown to consistently yield highly oxidized graphene oxide, as reported in our earlier studies.^37^ A very rigorous physicochemical characterization, including XPS, FTIR, Raman, and DSC, of both the initial GO and the GO–DES composites has been reported.

XPS was used to quantify the oxidation state (C/O ratio) and identify functional groups (e.g., hydroxyl, epoxy, and carbonyl). As calculated from the XPS survey spectrum reported in Figure S1­(a) and Table S1, the resulting quantitative estimate of the C/O ratio is about 2, showing a high degree of oxidation of the material (this ratio varies according to the synthetic procedure followed, and the oxidizing system chosen). The XPS C 1s core level spectrum presented in Figure S1­(b) reveals a predominant presence of hydroxyl and epoxy groups, accounting for approximately 48% of the carbon species, which is characteristic of GO produced via the Hummers’ method. Carbonyl groups were identified in lower abundance, contributing around 8% to the total C 1s signal, as detailed in Table S1.

The FTIR spectrum of GO, depicted in Figure S2, confirms the successful oxidation of the material in line with the findings in the literature.^57^ The distinct carbonyl absorption band at approximately 1739 cm^–1^, along with a shoulder at around 1815 cm^–1^ attributed to lactone vibrations, highlights the presence of fully oxidized material. Additional evidence of oxidation is provided by the contributions from alcohol groups, visible at 1368 cm^–1^ (C–OH bending of tertiary alcohols), 1281 cm^–1^ (C–OH bending of carboxyl groups), and 1066 cm^–1^ (C–OH stretching of tertiary alcohols). Furthermore, the presence of C–O–C stretching vibrations from epoxy functionalities is indicated by a shoulder near 985 cm^–1^ and a broad band between 840 and 750 cm^–1^.

The results obtained from XPS and FTIR analyses were employed to construct a representative GO sheet model for molecular dynamics simulations, as described in the previous section. To accurately reflect the structural features observed experimentally, holes were incorporated into the simulated GO plane. These structural defects are consistent with findings from previous studies^37^ and are attributed to the harsh oxidative conditions used during synthesis, which can partially degrade the carbon framework and introduce significant disruptions in the graphene lattice.

### Thermal Analysis of GO-DES by DSC

DSC was employed to characterize the thermal properties of GO–DES composites and their components, such as thermal stability and phase transitions. In Figure 1 are reported the DSC data of (a) choline chloride (blue line), (b) ethylene glycol (red line), (c) ethaline (violet line), (d) GO–ethaline (black line), and (e) GO (brown line), obtained by heating the samples from RT to 350 °C at 10 °C/min rate.

**Figure 1:**
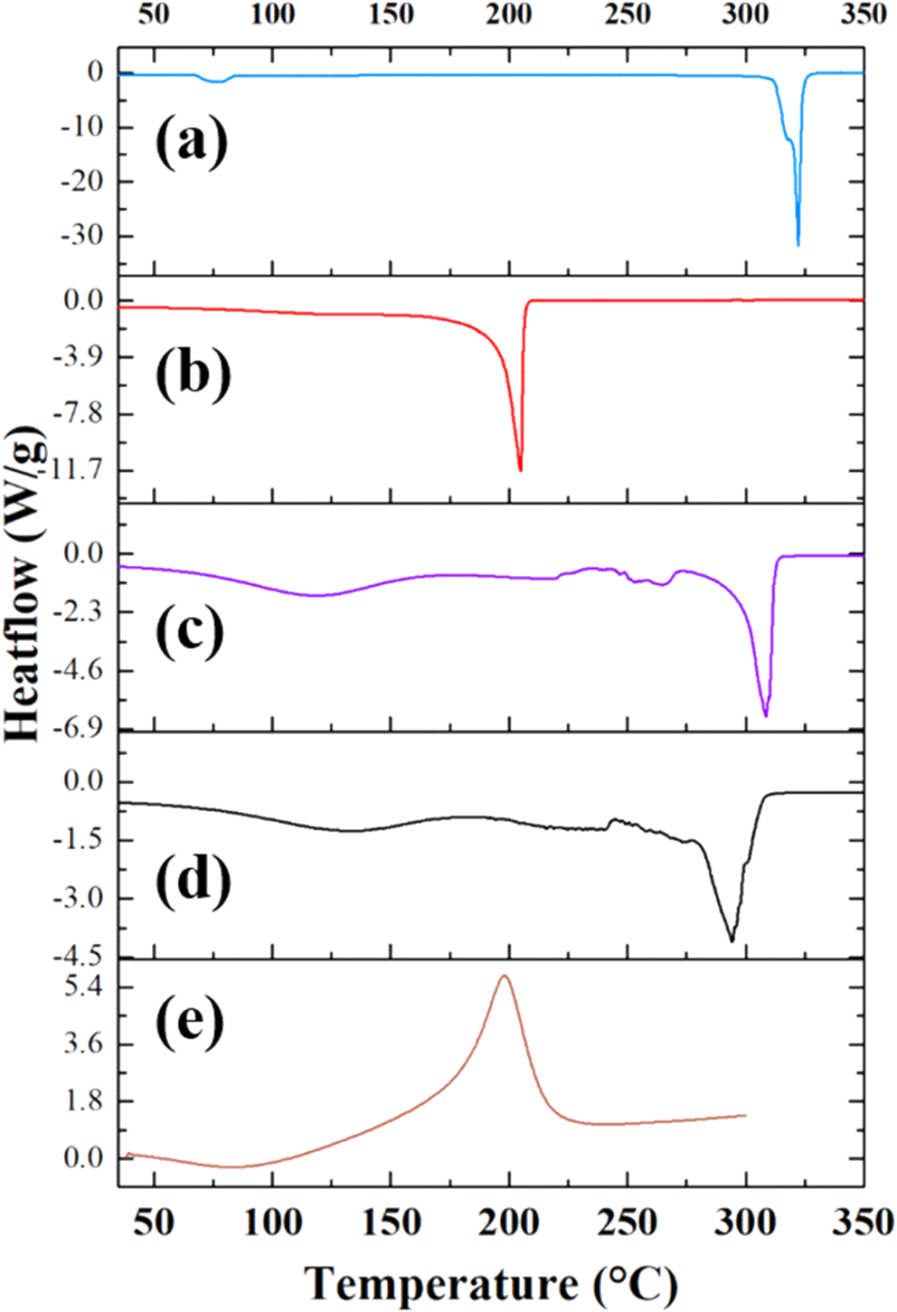
DSC of (a) choline chloride (blue line), (b) ethylene glycol (red line), (c) ethaline (violet line), (d) GO–ethaline (black line), and (e) GO (brown line). Heating segment from RT to 300–350 °C at 10 °C/min.

The DSC data analysis reveals interesting thermal features. Choline chloride (Figure 1a, blue line) typically shows a sharp endothermic peak in the DSC curve around 320 °C, which corresponds to its melting point. After melting, ChCl decomposes at higher temperatures without showing any crystallization peaks, indicating thermal instability above the melting temperature. EG shows endothermic peaks at 205 °C corresponding to its boiling point, after which it begins to decompose (Figure 1b, red line). Ethaline (Figure 1c, violet line) exhibits a broad endothermic peak centered at around 100 °C, attributed to the removal of moisture. This is followed by several endothermic peaks indicating melting, particularly at approximately 252 and 265 °C, where the DES fully melts. The most pronounced endothermic peak occurs at 310 °C, likely resulting from the decomposition of the ChCl component. The thermal behavior of the GO–ethaline composite (Figure 1d, black line) is similar to that of ethaline but with a notable shift in the decomposition peak to lower temperatures, around 294 °C. This shift can be attributed to interactions between GO and ethaline. In contrast, the thermogram of GO (Figure 1e, brown line) exhibits typical features, beginning with a broad endothermic peak around 90 °C due to the removal of moisture and labile oxygenated groups. This is followed by an exothermic peak centered at 200 °C, associated with the removal of the most oxygenated groups and exhibiting an enthalpy change of 756 J/g. Notably, the thermal analysis of the GO–ethaline composite does not show a distinct decomposition peak for GO. This may be due to the low percentage of GO in the mixture, which is less than 2%.

The thermal properties of DES and DES-GO samples can be further investigated by analyzing the DSC curves at low temperatures since DSC is able to identify phase changes, such as glass transitions and crystallization processes, which can occur at low temperatures. Within this aim, the GO–DES samples were first cooled from room temperature (RT) to −80 °C, kept in isothermal conditions for 5 min at −80 °C, and then heated back to RT at a controlled rate of 2 °C/min.

The thermal behavior of ethaline and GO–ethaline composite at low temperatures is reported in Figure 2. Observing the two DSC traces reported, it is noteworthy to observe that ethaline is not strongly affected by the cooling process, forming a supercooled phase, as no transitions occur in the investigated range. GO–ethaline composite curve, instead, shows an important exothermic transition at about −35 °C, originating from a cold crystallization transition. The presence of GO in the mixtures, although in small percentages, can strongly alter the thermal properties of the mixtures, enhancing the cold crystallization transitions, which are not observed in pure ethaline.

**Figure 2:**
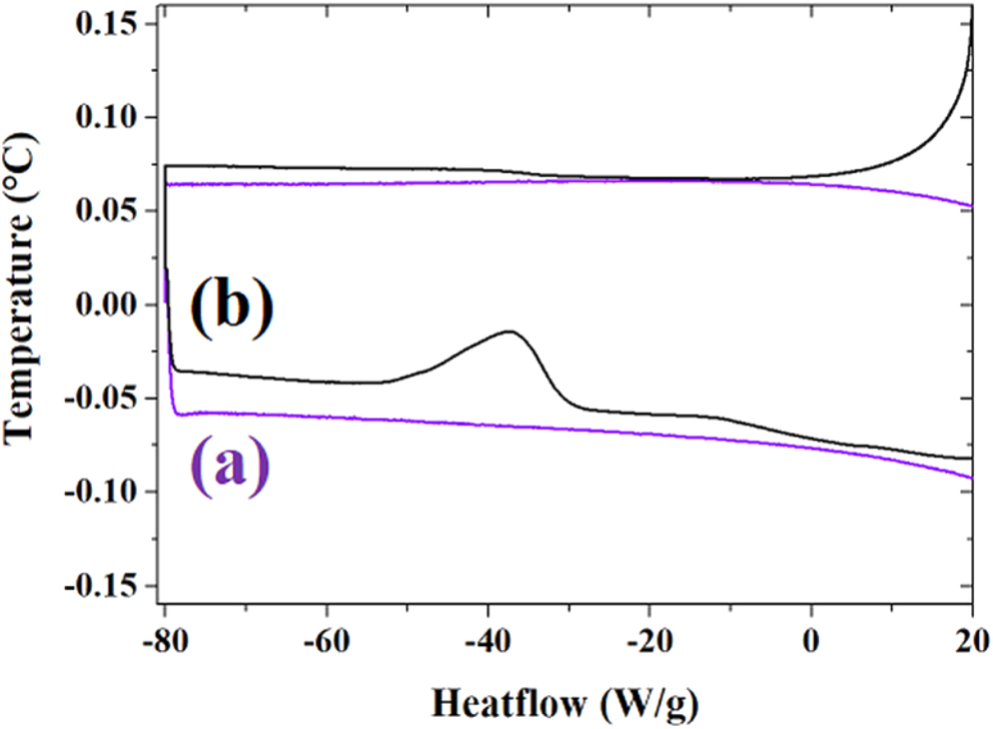
DSC of (a) ethaline (violet line) and (b) GO–ethaline (black line) at low temperature cycling. Cooling from RT to −80 °C, isothermal at −80 °C for 5 min, heating from −80 °C to RT, at 2 °C/min.

The thermal analysis was then carried out for the other GO–DES system, and Figure 3 shows the DSC data of (a) choline chloride (blue line), (b) urea (light green line), (c) reline (dark green line), (d) GO–reline (gray line), and (e) GO (brown line), obtained by heating the samples from RT to 350 °C at 10 °C/min rate.

**Figure 3:**
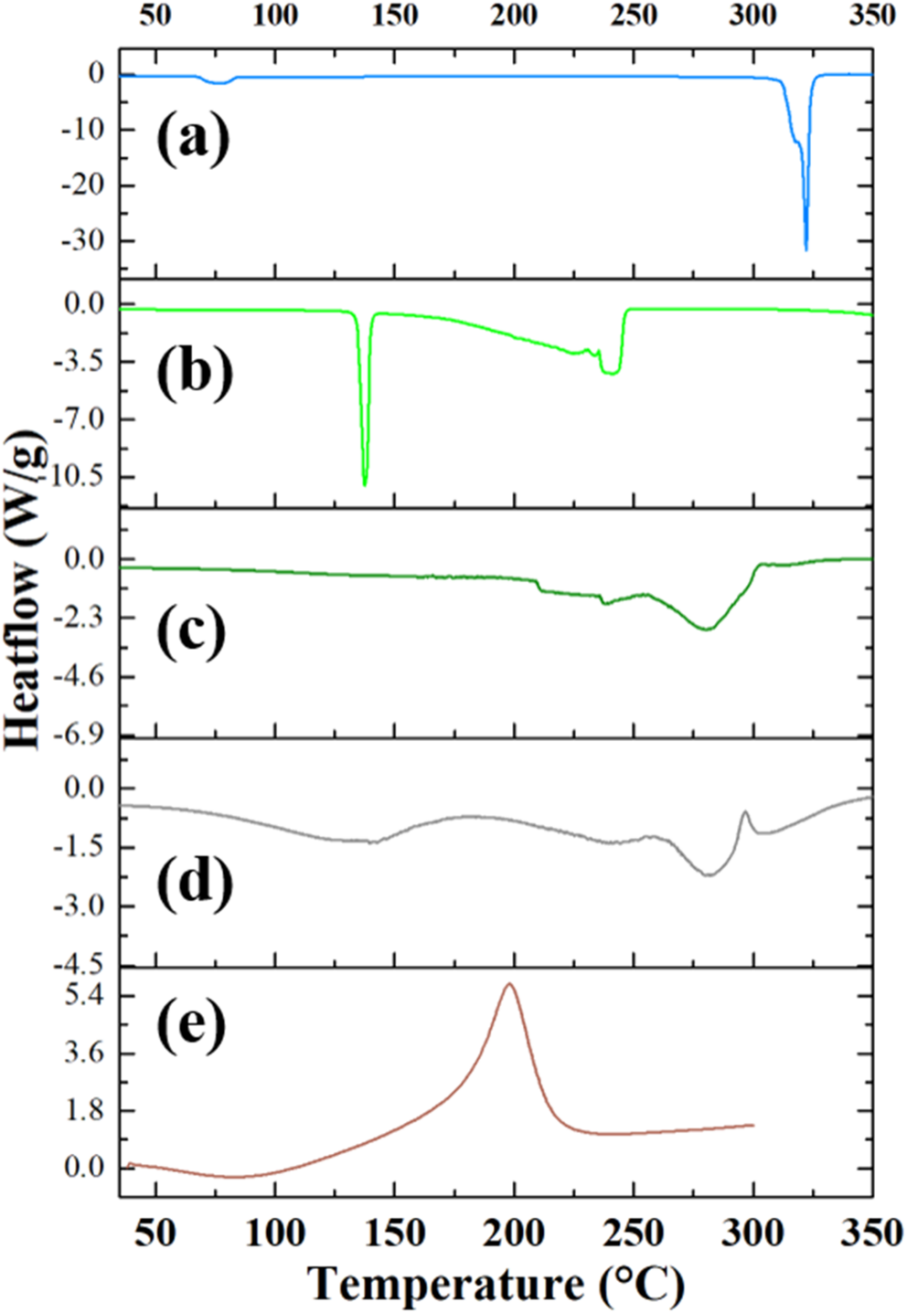
DSC of (a) choline chloride (blue line), (b) urea (light green line), (c) reline (dark green line), (d) GO–reline (gray line), and (e) GO (brown line). Heating segment from RT to 300–350 °C at 10 °C/min.

The urea curve (Figure 3b, light green line) shows a sharp endothermic melting peak at 140 °C, while the decomposition process starts at around 200 °C, as evidenced by a series of complex endothermic peaks. In contrast, reline (Figure 3c, dark green line) exhibits multiple endothermic transitions starting near 210 °C, with the main melting peak centered at 280 °C, indicating the complete melting of the deep eutectic solvents. The GO–reline composite (Figure 3d, gray line) exhibits a broad endothermic peak at 100 °C, attributed to the removal of moisture, followed by the same melting behavior of the DES alone. As for the GO–ethaline system, the thermal analysis of the GO–reline composite does not show a distinct decomposition peak for GO.

The thermal behavior of reline at low temperature appears strongly different from that of ethaline: in Figure 4, indeed, reline shows no transitions in the cooling process; on heating, the undercooled liquid formed during the cooling process undergoes a cold crystallization transition centered approximately at −35 °C. The solid phase formed melts with a broad transition at 2 °C. The DSC trace of the GO–reline composite shows an important cold crystallization transition at about −48 °C, 15° lower than reline. The composite does not exhibit any melting process, at least within the thermal range considered in these measurements. As for ethaline-based systems, also in this case, the presence of GO affects the thermal properties of the systems.

**Figure 4:**
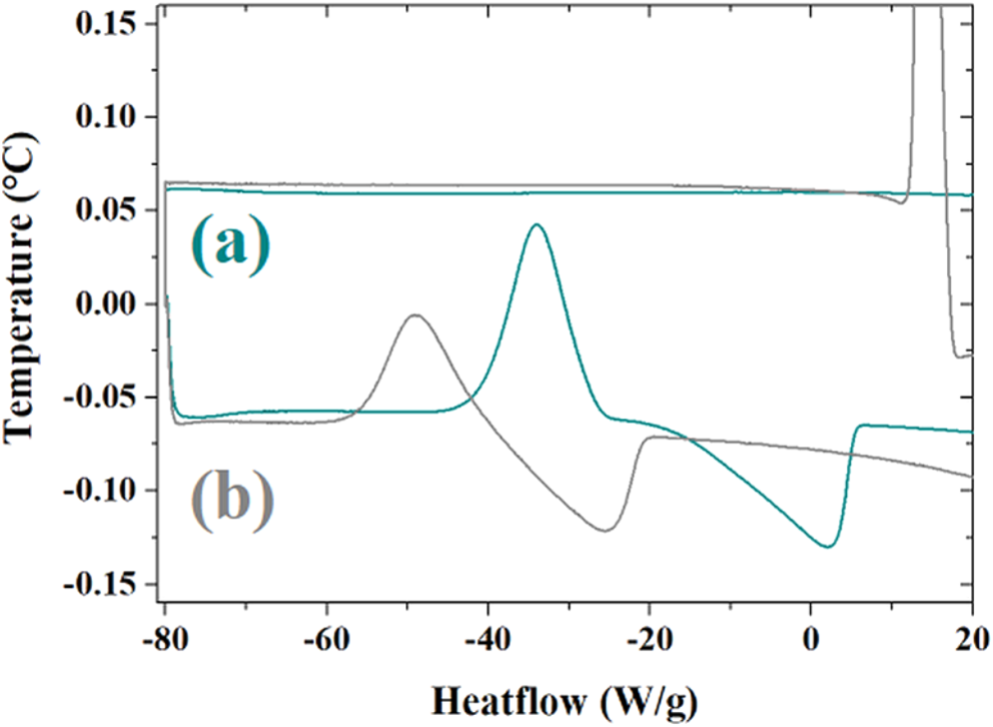
DSC of (a) reline (dark green line) and (b) GO–reline (gray line) at low temperature cycling. Cooling from RT to −80 °C, isothermal at −80 °C for 5 min, heating from −80 °C to RT, 2 °C/min.

### Infrared Spectra of DES and GO–DES Systems

Within the aim to identify the groups of DES and GO involved in interactions, infrared spectra were measured for each system starting from DES and its components and comparing their spectra with those of DES in the presence of GO vibrational spectroscopy identifies the nature of the functional groups in DES and GO (e.g., epoxy vs hydroxyl) and reveals the presence of intermolecular interactions between components in DES and components with GO, as, for example, hydrogen bonding.

Figure 5 shows the FTIR spectra of ethaline (choline chloride-ethylene glycol = 1:2) and its components before interaction with GO.

**Figure 5:**
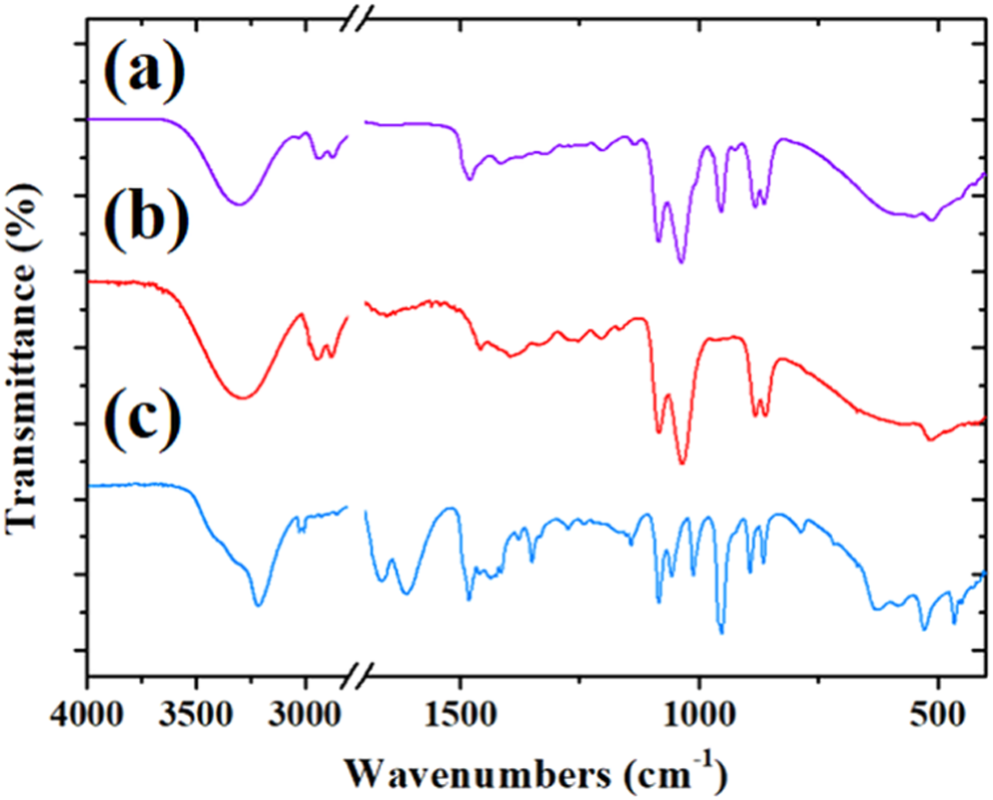
FTIR spectra of (a) ethaline (violet line), (b) ethylene glycol (red line), and (c) choline chloride (blue line).

The FTIR spectrum of ethaline (Figure 5a, violet line) exhibits several characteristic absorption bands indicative of the functional groups present in the DES constituents and their involvement in intermolecular interactions. The broad band observed at 3306 cm^–1^ in ethaline is related to the stretching vibration of the O–H groups present in both EG and ChCl components. A comparison with the OH stretching absorptions of the pure components shows that mixing causes small frequency shifts: for example, the OH stretching in EG is observed at 3294 cm^–1^. This slight shift toward higher wavenumbers can be attributed to the formation of hydrogen bonds between ethylene glycol and choline chloride and a weakening of the hydrogen-bond interactions between EG molecules in the bulk of the pure component.

Signals derived from ethylene glycol are present practically unchanged in the ethaline spectrum: those at 2937 and 2872 cm^–1^ are attributed to the C–H stretching vibrations, those at 1035 and 1084 cm^–1^ to the C–O stretching modes, while the absorptions at 882 and 863 cm^–1^ can be assigned to vibrations involving rocking CH_2_ modes and C–C stretching modes.^58^ Similarly, some signals observed in the choline chloride spectrum and previously^59^ assigned the bending CH_2_ modes (1480, 1320, and 1050 cm^–1^), C–O stretching (1135 cm^–1^), and C–N stretching (1000 cm^–1^) are still well identified in the ethaline spectrum.

The FTIR spectrum of the GO–ethaline composite is compared with those of GO and ethaline in Figure 6: (a) GO (brown line), (b) GO–ethaline (black line), and (c) ethaline (violet line). As expected, the FTIR spectrum of the GO–ethaline system, Figure 6b, typically displays several characteristic peaks that reflect the functional groups present in both components. For example, the broad peak around 3400 cm^–1^ is indicative of the stretching mode of the OH groups from both GO and ethaline. A quick comparison between the spectral region of these three systems shows that the absorption of the GO–ethaline composite is quite broader than those of GO and ethaline, suggesting that the interaction of GO with single components of DES could involve the OH groups. The C=O stretching peak observed at 1720 cm^–1^ in GO shifts to 1730 cm^–1^ in the GO–ethaline composite, suggesting an interaction between the carbonyl groups of GO and ethaline. The C=C stretching vibration is distinctly observed at 1598 cm^–1^ in the composite, but, unfortunately, this peak is obscured by the water bending vibration at 1620 cm^–1^ in the GO spectrum. The absorption at 1368 cm^–1^ in the GO–ethaline spectrum is assigned to the bending vibration of the alcohol group, consistent with its presence in the GO spectrum. Notable shifts are also observed for the carboxylic acid bending mode, which moves from 1280 cm^–1^ in GO to 1276 cm^–1^ in GO–ethaline, and the tertiary alcohol stretching, which shifts from 1040 cm^–1^ in GO to 1063 cm^–1^ in the GO–ethaline composite. Additionally, the stretching vibration of the epoxy group, observed at 845 cm^–1^ in GO, appears at 836 cm^–1^ in the GO–ethaline spectrum. All of these spectral shifts highlight the presence of significant interactions between the GO functional groups and the ethaline solvent.

**Figure 6:**
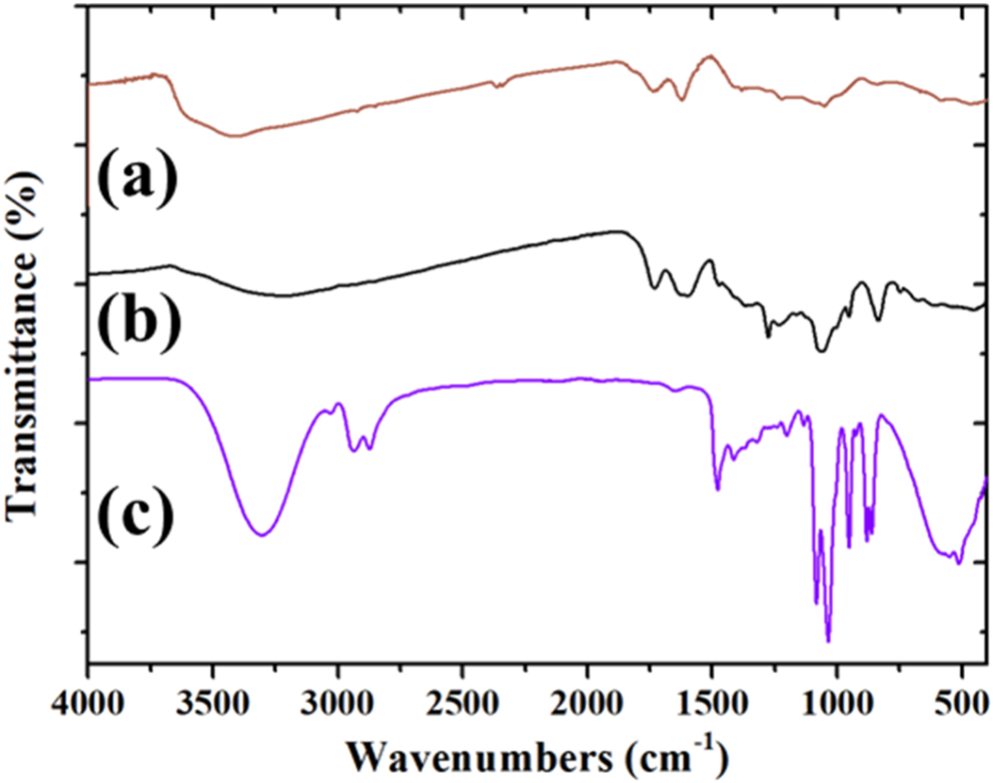
FTIR spectra of (a) GO (brown line), (b) GO–ethaline (black line), and (c) ethaline (violet line).

As for the GO–ethaline system, we analyzed the second GO–DES composite starting from the FT-IR spectra of reline and its components as reported in Figure 7.

**Figure 7:**
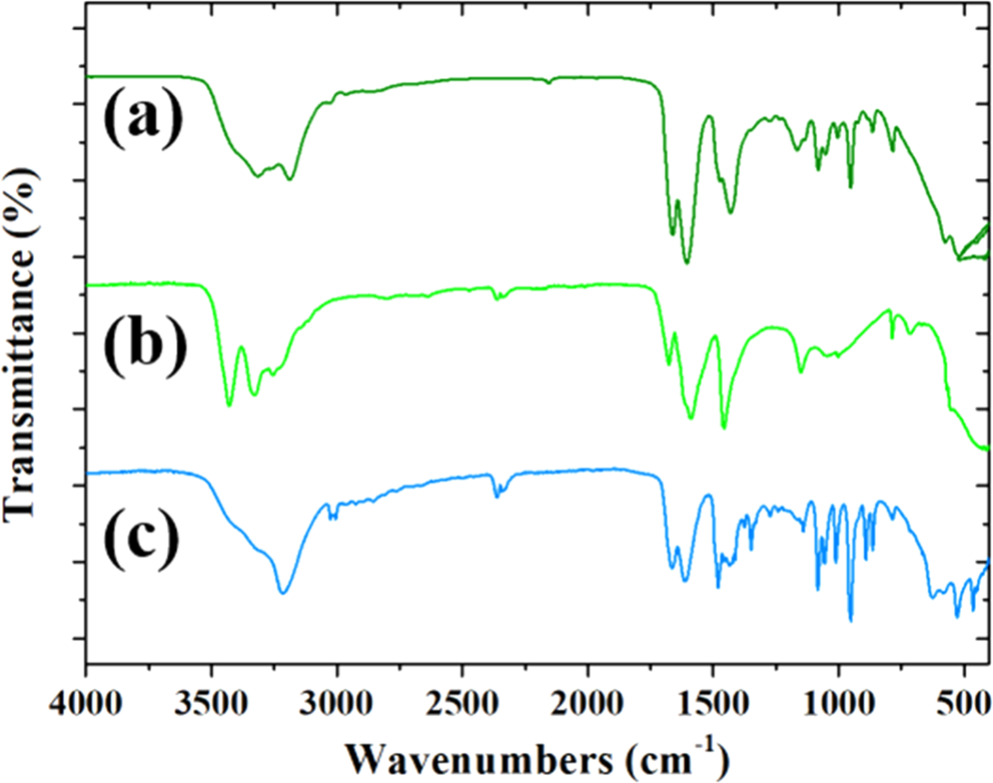
FTIR spectra of (a) reline (dark green line), (b) urea (light green line), and (c) choline chloride (blue line).

As expected, the FTIR spectrum of reline (Figure 7a, dark green line) reveals several absorption bands that reflect the nature of the functional groups of its components. For example, the high frequency spectrum of reline shows a broad band around 3300 cm^–1^, due to the overlap of the stretching modes of NH of urea and OH of choline, measured in the pure components at 3430 and 3201 cm^–1^, respectively. It is interesting to note that both the stretching modes are shifted upon mixing, revealing the formation of a strong hydrogen bonding within the DES. As for ethaline, the bands of the C–H stretching of choline are not significantly affected by mixing, whereas the signal of the C=O stretching of the CO-NH_2_ group shifts from 1678 cm^–1^ in urea to a lower frequency of 1668 cm^–1^ in reline. The shift indicates once again a strong interaction between urea and choline in the reline, likely due to hydrogen bonding. The formation of hydrogen bonds causes a weakening of the force constant of the normal vibrational coordinate, a consequent frequency red shift, and a simultaneous broadening of the absorption. Consistently, spectral changes are observed also for the N–H bending vibrations of urea (from 1150 cm^–1^ in pure urea to 1165 cm^–1^ in reline), confirming that hydrogen bonding in DES involves the NH group of urea. A notable peak around 600–800 cm^–1^ may be related to the bending vibrations of the O–H group involved in forming hydrogen bonds with the Cl ion of choline. The whole of the spectral changes observed by mixing the components is largely indicative of the complex hydrogen-bonding network that characterizes reline, highlighting the microscopic interactions between choline chloride and urea leading to the formation of this unique solvent system.

The FTIR spectra of the GO–reline composite and its components are presented in Figure 8: (b) GO–reline (gray line) and (c) reline (dark green line).

**Figure 8:**
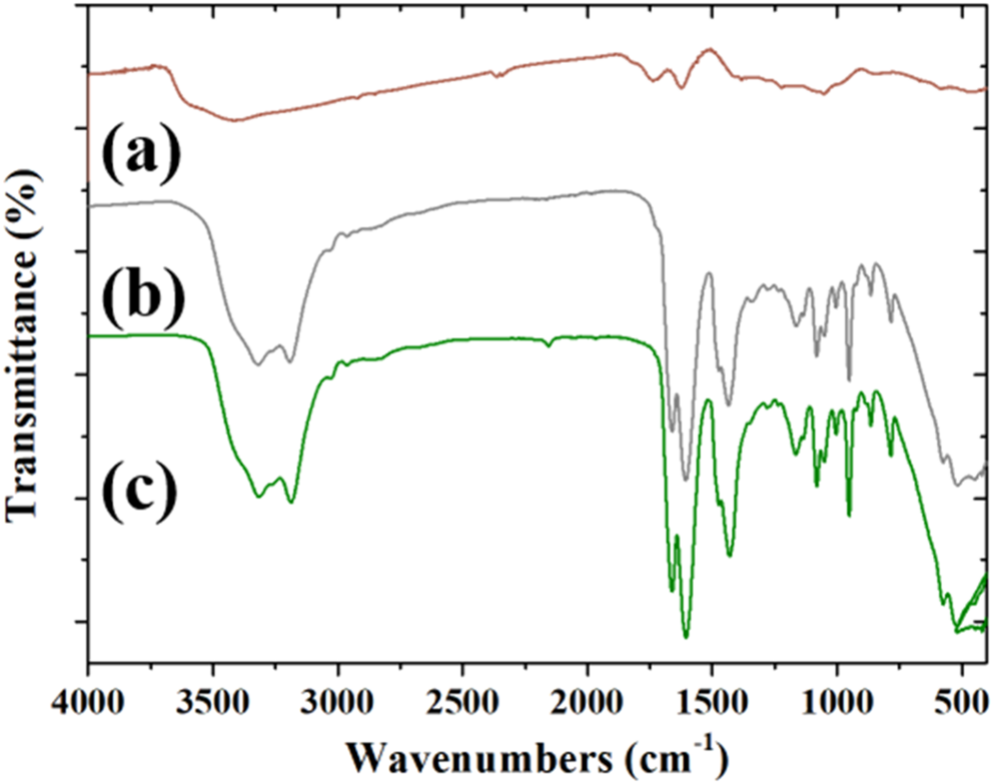
FTIR spectra of (a) GO (brown line), (b) GO–reline (gray line), and (c) reline (dark green line).

The spectrum of the GO–reline composite, shown in Figure 8b as a gray line, displays a broad double peak in the O–H/N-H region at 3323 and 3190 cm^–1^ as observed for reline, indicating that the strong hydrogen-bonding interactions present in DES continue to play a crucial role also in the presence of GO. Additionally, the presence of C=O stretching at 1720 cm^–1^, typical of the GO structure, appears in the composite spectrum as a shoulder to the stronger C=O stretching band of the CO-NH2 group at 1668 cm^–1^ in reline. In the 1500–800 cm^–1^ region, the most intense peaks characteristic of reline obscure the weak signals associated with GO, making it challenging to discern the specific contributions from GO. However, in the spectrum of the GO–reline composite, small shifts are observed in the reline signals compared to those of pure reline, suggesting possible interactions with GO. Specifically, the peak at 1607 cm^–1^ in pure reline shifts to 1605 cm^–1^ in the composite, one at 1430 cm^–1^ moves to 1435 cm^–1^, and the absorption at 1346 cm^–1^ lowers to 1339 cm^–1^, suggesting slight alterations in the hydrogen-bonding or bonding environment in the presence of GO.

### Raman Spectra of DES and GO–DES Systems

Raman spectroscopy is a widely used method to characterize carbon structures, capable of estimating the structural features of the materials themselves, as it brings specific information on the presence and relative quantification of defects. The Raman spectra of GO–ethaline (a), ethaline (b), and GO (c) are compared in Figure 9, whereas in Figure S3, we report the Raman spectra of ethaline (Figure S3a) and its component ethylene glycol (Figure S3b) and choline chloride (Figure S3c).

**Figure 9:**
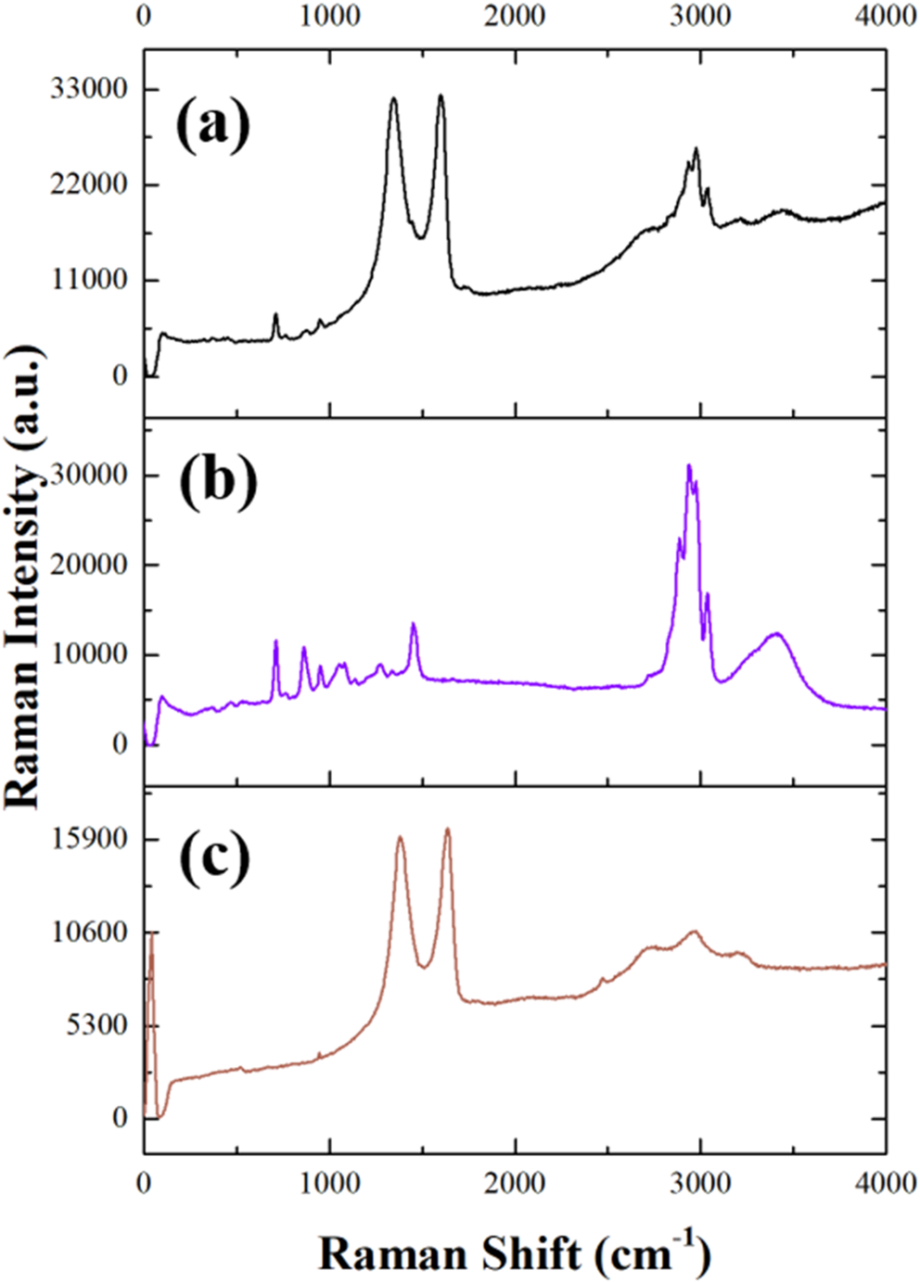
Raman spectrum of (a) GO–ethaline (black line), (b) ethaline (violet line), and (c) GO (brown line).

The GO spectrum, Figure 9c, has the characteristic shape of graphene-based materials, with two peaks at 1385 and 1635 cm^–1^, which are assigned, respectively, to the D-band related to the presence of defect sites (vacancies, grain boundaries, and edges) and the G-band, which is characteristic of the graphene skeleton.^60^ The intensity ratio of the D and G bands (*I*_D_/*I*_G_) is used to estimate the number and size of the sp^2^ domains and may be considered as an indirect estimation of the disorder within the GO material. The D-band peak indicates structural imperfections induced by the presence of hydroxyl and epoxide groups on the carbon basal plane. The Raman spectra confirm the introduction of a considerable amount of structural disorder in the graphene lattice due to the oxidation process, and the *I*_D_/*I*_G_ ratio is 0.91.

The Raman spectrum of the GO–ethaline composite, Figure 9a, shows again the two characteristic intense GO bands, shifted to lower wavenumbers: 1345 cm^–1^ for the D band and 1605 cm^–1^ for the G band, respectively. The presence of ethaline in the composite is confirmed by the sharp and weak Raman peak at about 710 cm^–1^, which corresponds to the C–N stretching of the quaternary ammonium group. Additionally, the complex peak centered at 3000 cm^–1^ that corresponds to the C–H stretching modes further verifies the presence of ethaline in the composite material.

When GO is mixed with reline, the Raman spectrum shown in Figure 10a (gray line) exhibits distinct peaks that reflect the presence of both GO and reline. The Raman spectra of reline (Figure S4a, dark green line) and its component urea (Figure S4b, light green line) and choline chloride (Figure S4c, blue line) are described in the Supporting Information. As observed for the GO–ethaline system, the two characteristic intense bands of GO are shifted to lower wavenumbers upon interaction with reline. Specifically, the D band shifted from 1385 to 1349 cm^–1^, while the G band shifted from 1635 to 1612 cm^–1^. These shifts suggest alterations in the electronic environment of GO, likely due to interactions with reline within the composite.

**Figure 10:**
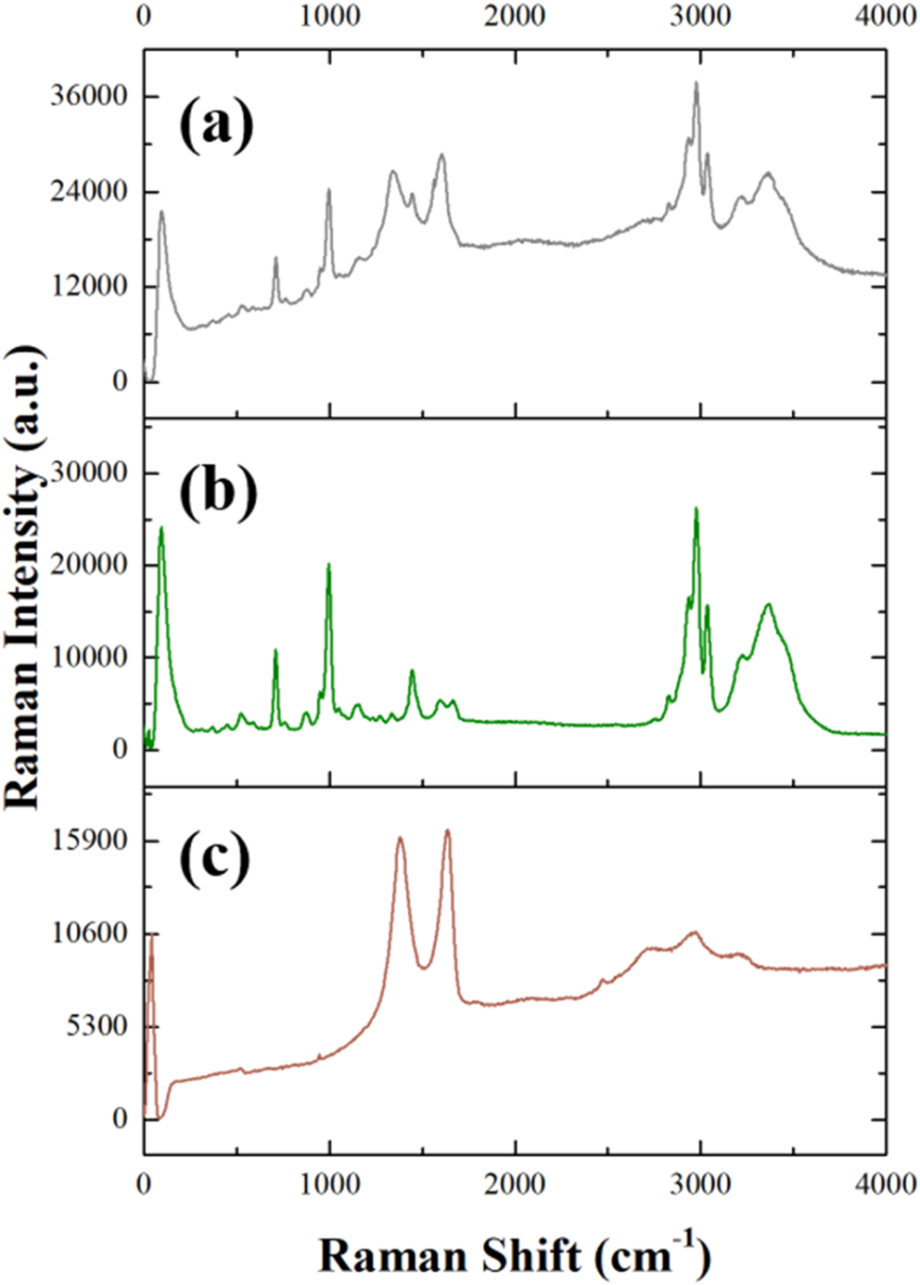
Raman spectra of (a) GO–reline (gray line), (b) reline (dark green line), and (c) GO (brown line).

### Computational Results

The systems were investigated by means of classical molecular dynamics simulation (MD) in order to simulate the interactions between ethaline and reline with the GO sheet in their respective bulk phases. The calculation of radial distribution functions, *g*(*r*), for some characteristic distances allowed us to strengthen and confirm the structural hypotheses obtained by analyzing the results of vibrational spectroscopy.

In Figure 11, we report the radial distribution functions computed for reline–GO composite systems.

**Figure 11:**
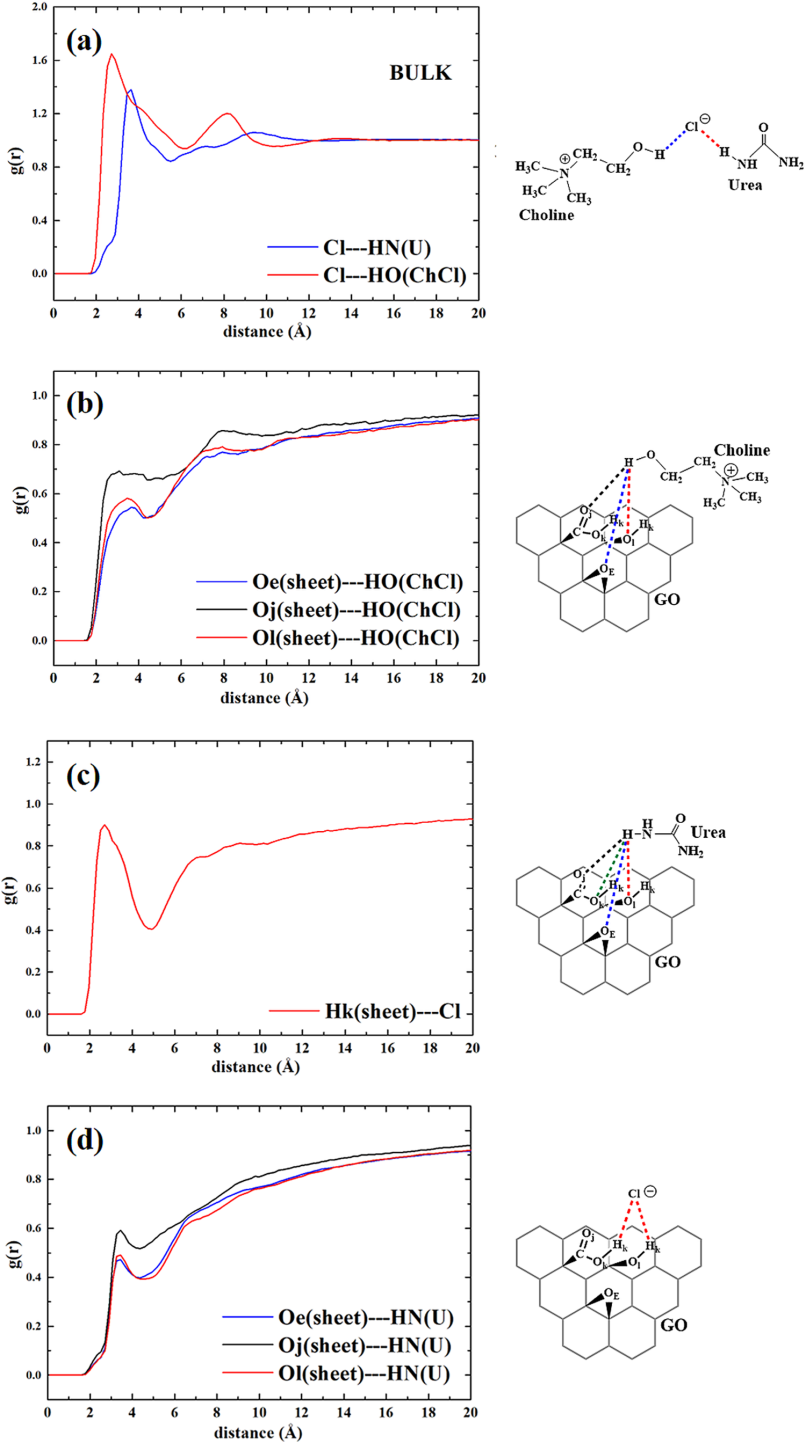
Radial distribution functions obtained for GO–reline composite and their graphical representation: (a) distances between chloride, urea, and choline; (b) distances between Cl and oxygenated groups of GO; (c) distances between OH (ChCl) and oxygenated groups of GO; and (d) distances between NH (urea) and oxygenated groups of GO.

The structural arrangement between ChCl and urea was investigated through the calculation of radial distribution functions, and the results are reported in Figure 11a. The coordination between choline and the anion could occur through two interaction sites: either by the positively charged nitrogen head or by hydrogen bonding with the terminal OH group. Several studies have shown that the latter mode of coordination is preferred. In our systems, a structured Cl···H­(OH) coordination peak is observed at around 2.2 Å, consistent with a possible hydrogen bond,^59^ as also observable in Figure S6, which indicates that short Cl···H distances are frequently associated with O–H···Cl arrangements close to linearity.

Urea coordinates chloride via the hydrogen atoms of its amide (NH) groups with a relatively broad distribution centered around 3.0 Å, as shown in the blue curve of Figure 11a. These distances are slightly longer than those typically reported for hydrogen bonds in pure DESs, possibly due to the influence of the GO sheet. This discrepancy can be rationalized by considering that, although reline is the dominant component in the GO–reline composite system, the presence of the GO sheet may perturb the strength and specificity of interactions between hydrogen-bond donor and acceptor groups, even for those not directly interacting with the surface. Nonetheless, the angular–radial distribution shown in Figure S6 reveals a clear correlation between short Cl···H distances and linear N–H···Cl arrangements, consistent with the geometry expected for hydrogen bonding. This suggests that although possibly weakened by long-range electrostatic effects induced by the GO sheet, directional interactions resembling hydrogen bonds still persist between urea and chloride. It is nonetheless noteworthy that, although the interactions occur at slightly larger distances than those typically found in pure liquids, the combined distribution function analysis (Figure S7) reveals that chloride often adopts configurations consistent with simultaneous hydrogen bonding to both urea and choline. This supports its role as a bridging hydrogen-bond acceptor in the DES environment. The interaction between the liquid components (choline, chloride, and urea) and the graphene sheet was then investigated. The distances between the chloride and the hydroxyl and acidic protons on the GO sheet (Hk) were analyzed to investigate the tendency of the sheet to act as HBD. As shown in Figure 11b, a noticeable peak is observed at approximately 2.5 Å, indicating that chloride can coordinate with the hydroxyl and acidic groups on the graphene sheet. It is interesting to observe that the oxygenated substituents on the graphene sheet can enable it to act as both a hydrogen-bond donor and an acceptor. Figure 11c shows the distances between all oxygenated groups and the hydroxyl proton of choline to explore whether the cation coordinates with the oxygen groups on the sheet through hydrogen bonding. Broad and low-intensity peaks are observed at approximately 2.6 Å between the hydroxyl proton of choline and both epoxide oxygen (Oe) and carboxylic oxygen (Oj). This suggests that choline, already coordinating with chloride, plays a limited role as a HBD toward the GO sheet. In contrast, the same distances measured between urea (NH groups) and the oxygenated groups of the sheet reveal the presence of more pronounced interactions at approximately 2.5–2.6 Å with all oxygen groups (epoxide, hydroxyl, and carboxylic acid). As a bidentate HBD, urea can simultaneously coordinate with chloride to form the DES and donate hydrogen bonds to the oxygenated groups on the GO sheet. However, it is important to note that the peaks in the radial distribution functions (*g*(*r*)) are weak, reflecting the low statistical probability of interactions between DES and GO, given the presence of only a single graphene sheet.

In the case of the GO–ethaline composite system, the RDFs indicate comparable distances between the chloride anion and the hydrogen-bond donor groups. In particular, the main peak of the RDF between the hydroxyl group of choline and chloride (Figure 12a) is centered at 2.25 Å, suggesting a close interaction compatible with hydrogen bonding. A second peak at 2.8 Å corresponds to the interaction between chloride and the hydroxyl groups of ethylene glycol, pointing to the possibility of H-bond formation with both components of the DES. Although these distances alone do not definitively prove hydrogen bonding, the angular-radial combined distribution analysis (Figure S5) reveals that the highest-probability regions correspond to geometries consistent with H-bond formation (i.e., short Cl···H distances and high O–H···Cl angles). This supports the presence of directional interactions in the liquid structure, even if they are possibly perturbed by the GO surface. Regarding coordination with the GO sheet (Figure 12b), a pronounced peak is observed between chloride and the hydroxyl groups on GO at 2.7–2.8 Å, indicating that chloride can also be effectively stabilized by surface oxygenated moieties, such as alcohols and carboxylic acids. This supports the view that Cl^–^ maintains its role as a hydrogen-bond acceptor not only within the DES matrix but also in interactions with the graphene oxide surface. As for the distances between the graphene sheet and choline, we observed a slightly different pattern compared to the previously described system. Specifically, as shown in Figure 12c, there is a relatively intense peak corresponding to the interaction between the OH group of choline and the sp^3^ oxygen of the carboxylic acid group, at approximately 3 Å (green curve). At the same distance but with lower intensity, an interaction is observed between the OH group of choline and the hydrogen group of the alcoholic substituents. From the comparison between the two systems, we could conclude that choline seems to be more efficiently coordinated with the GO sheet in ethaline than in reline. On the other hand, examining the curves shown in Figure 12d, the coordination between ethylene glycol and the sheet seems to be less efficient, as indicated by low-intensity peaks corresponding to interactions between the alcoholic protons of glycol and the oxygenated substituents present on the GO sheet, occurring at a distance of approximately 3 Å.

**Figure 12:**
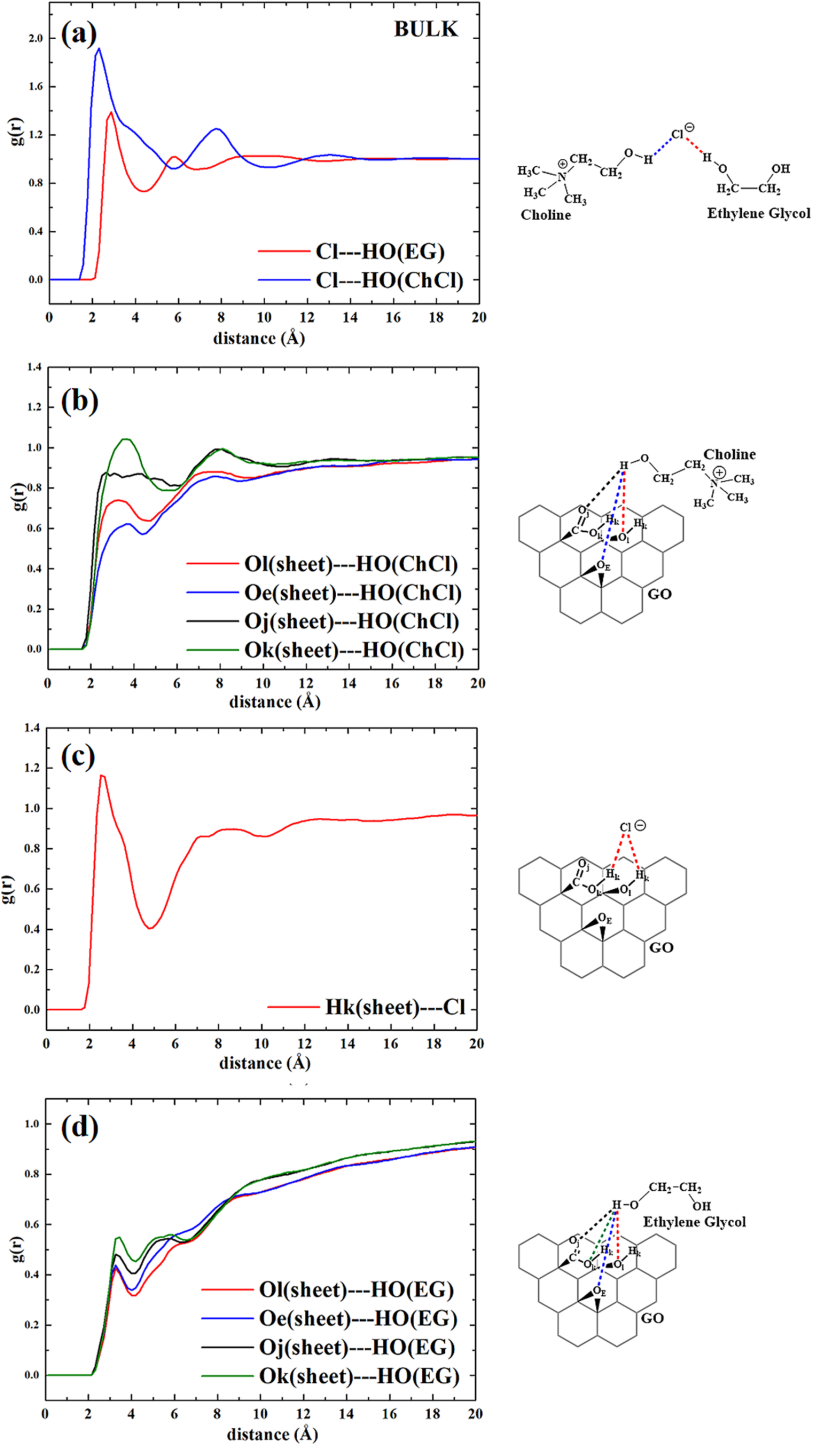
Radial distribution functions obtained for ethaline–GO composite and their graphical representation: (a) distances between chloride, ethylene glycol, and choline; (b) distances between Cl and oxygenated groups of GO; (c) distances between OH (ChCl) and oxygenated groups of GO; and (d) distances between OH (EG) and oxygenated groups of GO.

It is worth noting that, in both systems, the GO sheet placed at the center of the simulation box was arranged in the flattest possible configuration, despite defects, holes, and oxygenated groups limiting the perfect planarity typically observed in unmodified graphene sheets. During the simulations, the sheets exhibited a tendency to form corrugated and less planar structures, indicative of efficient coordination between the liquid groups and the GO.

## Conclusions

A multitechnique characterization approach (combining XPS, Raman, FTIR, and DSC) ensures that MD models accurately replicate GO’s chemical heterogeneity and dynamic behavior. A rigorously parametrized GO model is essential for reliably predicting interactions with deep eutectic solvents (DES), where the spatial distribution of functional groups and the density of structural defects critically influence solvent structuring and interfacial reactivity.

The surface chemistry of GO, particularly the nature and distribution of oxygen-containing groups, plays a central role in determining its interaction with DES. GO’s reactivity can vary significantly between aqueous environments and DES systems, owing to possible structural transformations such as the conversion of epoxides into hydroxyl groups. These changes affect the hydrogen-bonding capabilities at the GO–DES interface. In particular, hydroxyl groups tend to promote possible hydrogen bonding (like ethylene glycol in ethaline) and enhance interfacial hydrophilicity, while epoxides may disrupt the hydrogen-bonding network (e.g., choline chloride-urea in reline) and reduce solvent compatibility. DESs such as ethaline and reline exhibit complex interaction networks involving choline chloride and species like urea or ethylene glycol with structural features consistent with hydrogen bonding between choline chloride and the hydrogen-bond donors. The introduction of graphene oxide (GO), which is rich in oxygenated functional groups, perturbs these delicate interactions. For example, choline ions may electrostatically bind to carboxylic acid groups (COOH) on the GO surface, disrupting the native coordination environment of chloride anions and decreasing ion mobility.^61^ In reline, urea molecules can form interactions consistent with H-bonding with GO hydroxyl groups, effectively anchoring the DES structure to the GO surface.^62^ Conversely, in ethaline, ethylene glycol preferentially interacts with epoxy groups on GO, which weakens the original glycol–choline interactions and promotes better GO dispersion.^63^

This study demonstrates that understanding and simulating these interfacial phenomena are essential for rationally engineering GO–DES systems with tailored properties. At the molecular level, the interplay between GO functional groups and DES components is pivotal in tuning key physicochemical parameters, including viscosity, ionic conductivity, and colloidal stability, properties that are critical for advanced applications in catalysis, energy storage, and environmental remediation.

By combining experimental characterization with molecular dynamics simulations, this work establishes a validated framework for elucidating GO–solvent interactions. These insights are broadly applicable, informing the design of next-generation functional materials across diverse fields such as coatings, membranes, and 3D-printed devices where control over interfacial chemistry is paramount. The findings provide practical guidance for selecting solvents and surface chemistries in hybrid nanomaterial development, supporting the creation of tunable, ecofriendly, and high-performance systems for both experimental and theoretical researchers.

## Supplementary Material



## Notes

Synthesis procedures for deep eutectic solvents (DES) and GO–DES composites; comprehensive characterization of the starting graphene oxide (GO), including X-ray Photoelectron Spectroscopy (XPS) and Fourier-Transform Infrared Spectroscopy (FTIR), along with the Raman spectra of the DES, ethaline, reline, and their components. Combined distribution functions (CDFs) are reported in the SI (PDF)
