# Supplementary material for: Deep Eutectic Solvent Interaction with Graphene Oxide: A Combined Experimental and Molecular Dynamics Characterization
Source: J Phys Chem B. 2025 Aug 26;129(36):9206–18. doi: 10.1021/acs.jpcb.5c03461 (PMC12434660; doi:10.1021/acs.jpcb.5c03461)
Supplement: Supplementary file 1 [file jp5c03461_si_001.pdf]

Supporting Information file:

Deep Eutectic Solvents Interaction With Graphene Oxide: A Combined Experimental And Molecular Dynamics Characterization.

Simone Di Muzio[a,b], *Fabio Ramondo*[c] *and Giulia Fioravanti*[d*]

[a] Istituto di Fotonica e Nanotecnologie, Consiglio Nazionale delle Ricerche, P.zza Leonardo da Vinci 32, Milan, 20133, Italy

[b] Istituto dei Sistemi Complessi, Consiglio Nazionale delle Ricerche, P.le Aldo Moro 7, Rome, 00185, Italy

[c] Department of Chemistry, Sapienza Università di Roma, P.le Aldo Moro 5, Rome, 00185, Italy

[d] Department of Physical and Chemical Sciences, University of L'Aquila, Via Vetoio, L'Aquila, 67100, Italy

[*]Corresponding author: giulia.fioravanti@univaq.it

Materials and methods

1. Synthesis of DES

1.1 Ethaline

1.2 Reline

2. Synthesis of GO-DES

3. X-Ray Photoelectron Spectroscopy (XPS) of GO

4. Fourier-Transform Infrared Spectroscopy (FTIR) of GO

5. Raman Spectroscopy (RS) of DES and components

6. Combined Distribution Functions (CDFs)

References

Materials and methods

1. Synthesis of DES

Choline chloride (C5H14ClNO, ≥98%, Product N. C1879), ethylene glycol (C2H6O2, ReagentPlus ≥99%, Product N. 102466) and urea (CH4N2O, ACS reagent 99-100%, Product N. U5128) were purchased from Sigma Aldrich (St Louis, MO).

1.1 Ethaline

Ethaline was prepared by mixing choline chloride (ChCl) and ethylene glycol (EG) in a molar ratio of 1:2 under an inert atmosphere. The mixture was stirred and heated to 80°C until a homogeneous, colorless liquid formed. The resulting ethaline was then stored at room temperature in a sealed bottle over molecular sieves to remove any moisture.

1.2 Reline

Reline was prepared by mixing choline chloride (ChCl) and urea (U) in the 1:2 molar ratio under an inert atmosphere. The mixture was heated at 80 °C with continuous stirring until a homogeneous colourless liquid was formed. The resulting reline was then stored at room temperature in a sealed bottle over molecular sieves to remove any moisture.

2. Synthesis of GO-DES

GO was prepared as described elsewhere [1], ensuring a consistent and reliable source through the preparation method and sample cleanup, which is critical for subsequent research involving GO-DES

composites. GO powder was placed in an oven overnight at 60 °C to remove its moisture content. 200 mg of GO were dispersed in 7 ml of DES using a tip sonicator (UP100H Sonicator Hielscher, 100W, 30kHz) at a pulse mode (power = 35%, on = 30 s, off = 5 s), followed by stirring for 2 hours at 65 °C under an inert atmosphere [2].

3. X-Ray Photoelectron Spectroscopy (XPS) of GO

XPS spectra were collected in ultra-high vacuum (UHV) conditions with a PHI 1257 (Perkin Elmer, USA) spectrometer, equipped with a monochromatic Al Kα source (hν=1486.6 eV) with a pass energy of 11.75 eV, corresponding to an experimental resolution of 0.25 eV. The acquired XPS spectra were fitted with Voigt line shapes and Shirley backgrounds. The samples were directly deposited on a Si substrate (1 cm^2, SiO_2, oxide thickness 270 nm). The substrate was previously sonicated 10 min with acetone (Sigma Aldrich 24201, >99%), 10 min with isopropyl alcohol (Sigma Aldrich W292907, 99.7%), and 10 min with diluted basis Piranha (1:3:4 of H_2O_2:NH_4OH:H_2O) (Hydrogen peroxide, Sigma Aldrich 95294, 30%; Ammonia solution, Sigma Aldrich 05002, 33%), and then finally rinsed by Milli-Q water. The GO sample was deposited by drop casting a dilute aqueous solution (1.0 mg/ml, volume of 20 μl).

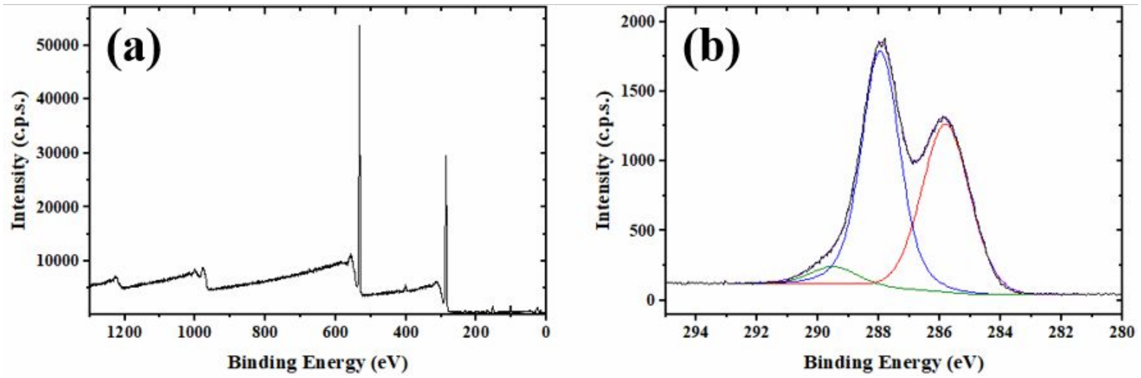

Figure S1. XPS (a) survey and (b) C1s region of GO.

The XPS survey spectrum shown in Figure S1(a) reveals the presence of only carbon and oxygen, with respective atomic percentages of 64.6% and 32.4%, as detailed in Table S1. The XPS C 1s core level spectrum is displayed in Figure S1 (b). The spectrum was fitted by the sum of three components assigned to C atoms belonging to: aromatic rings and hydrogenated carbon (C=C/C-C, 284.8 eV),

hydroxyl and epoxy groups (C-O/C-O-C, 286.9 eV) and carbonyl groups (C=O, 288.2 eV). The relative abundances of each component of the C1s spectra are: C=C/C-C 44.7%; C-O/C-O-C 47.7%; C=O 7.6%, as reported in Table S1.

**C 1s survey (284.8 eV)**	**O 1s survey (532 eV)**	**C-C/C=C (284.8 eV)**	**C-OH/C-O-C (285.9 eV)**	**C=O/COOH (288.2 eV)**
64.6	32.4	44.7	47.7	7.6

Table S1. XPS data of GO sample, with atomic percentage of C 1s and O 1s, and C 1s components.

4. Fourier-Transform Infrared Spectroscopy (FTIR)

The functional groups of the samples were analysed with FTIR spectroscopy. The samples were dried in an oven, for at least 24 hours at 60 °C, and then directly characterized. FTIR spectra were performed at RT at wavelength 4000-400 cm^{-1} on a Perkin Elmer spectrophotometer Spectrum Two, equipped with reflectance module (ATR) at 2 cm^{-1} spectral resolution, with the accumulation of 16 repeated scans.

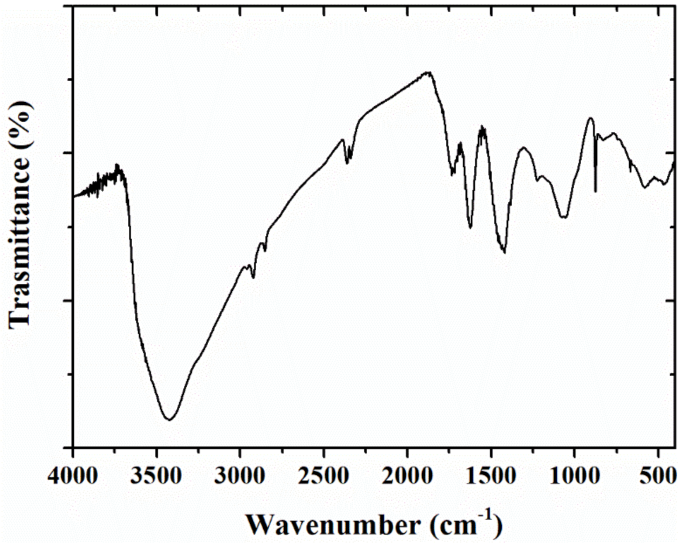

Figure S2. FTIR spectrum of GO

The GO FTIR spectrum, reported in Figure S2, showed a broad band at approximately 3420 cm^{-1}, corresponding to the stretching vibrations of the O-H bond. The bands at 2926 cm^{-1} and 2850 cm^{-1} are attributed to the stretching vibrations of CH and CH_2 groups associated with sp^3 carbon. The

absorption band at 1739 cm^{-1} is due to the stretching vibrations of C=O from carbonyl or conjugated carbonyl groups. Additionally, a shoulder peak at 1815 cm^{-1} suggests the presence of lactone stretching. The peak at 1580 cm^{-1} assigned to the C=C stretching of aromatics overlaps with a bending band of water observed at 1620 cm^{-1}. Further peaks around 1368 cm^{-1}, 1281 cm^{-1}, and 1066 cm^{-1} are linked to C-OH bending from tertiary alcohols, C-OH bending from carboxyl groups and C-OH stretching from tertiary alcohols, respectively. Lastly, C-O-C stretching from epoxy or ether groups is indicated by a shoulder around 985 cm^{-1} and as broad band between 840-750 cm^{-1}.

5. Raman Spectroscopy (RS)

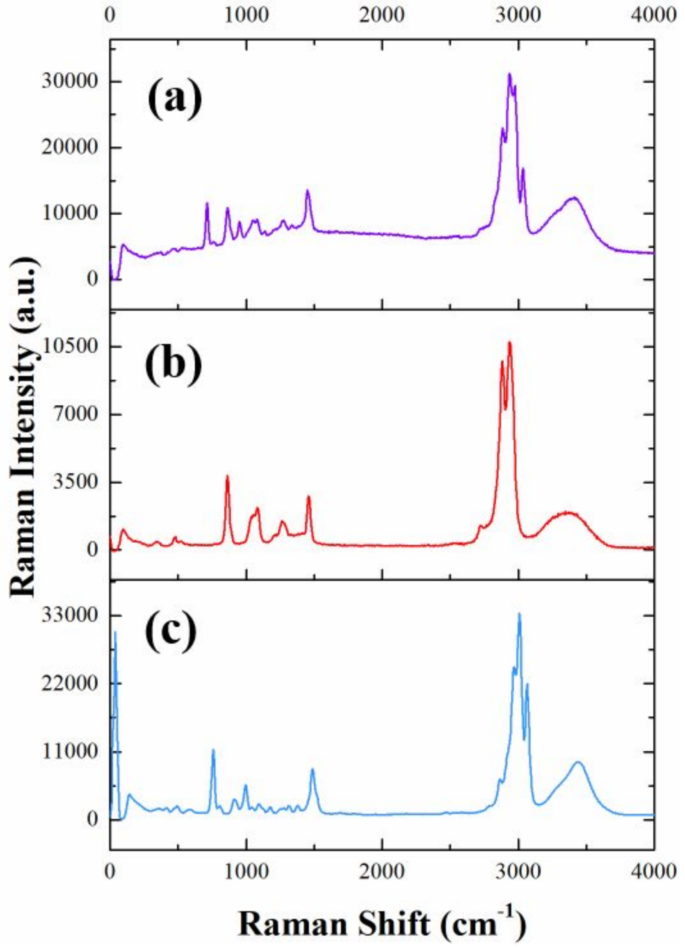

Figure S3. Raman spectrum of (a) Ethaline (violet line), (b) Ethylene Glycol (red line) and (c) Choline Chloride (blue line).

The Raman spectrum of ethaline, reported in Figure S3, would likely exhibit the combined vibrational modes of both its components. Consistently with the IR spectrum, a subtle shift of the broad O–H stretching absorption in the DES spectrum (3400 cm^{-1}) suggests the presence of hydrogen bonding. In the region of 2800-3050 cm^{-1}, overlapping features from both constituents result in a complex spectrum, corresponding to the C–H stretching modes. In the spectrum of ethaline some peaks can be attributed to the EG, such as the peaks at 1080 cm^{-1} and 860 cm^{-1} which correspond to the C–O and C–C stretching vibrations, respectively. These are associated with the stretching vibrations of the carbon backbone in ethylene glycol, reflecting its contribution to the overall vibrational profile of the ethaline system. The strong peak near 760 cm^{-1}, corresponding to the C–N stretching of the quaternary ammonium group in ChCl component, was shifted to lower wavenumbers into ethaline (710 cm^{-1}).

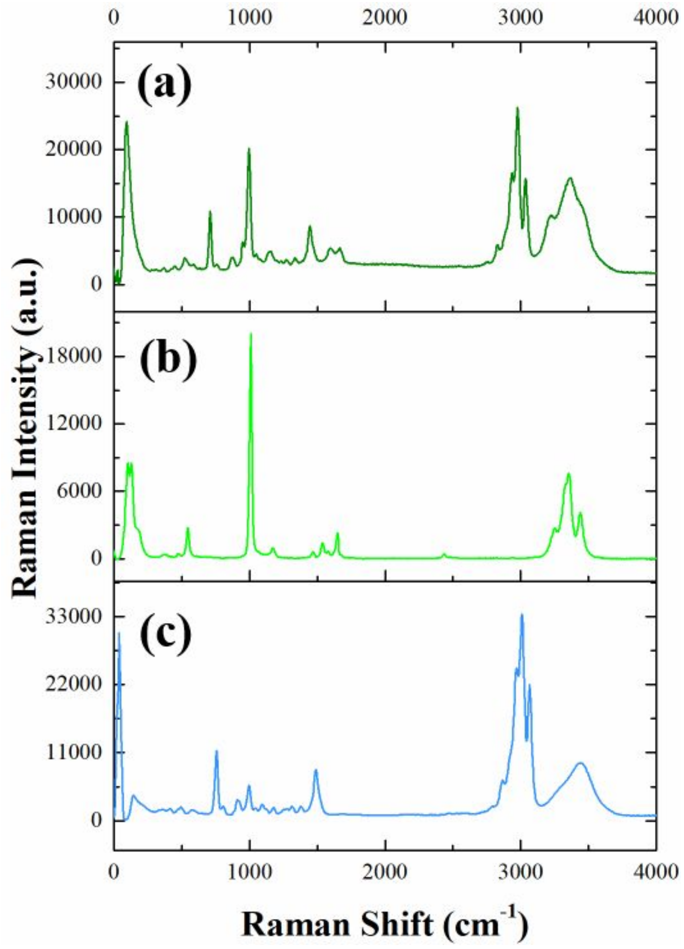

Figure S4. Raman spectra of (a) reline (dark green line), (b) urea (light green line) and (c) choline chloride (blue line)

The Raman spectra of reline and its components are reported in Figure S4. The broad vibration at 3370 cm^{-1} is related to O–H stretching signal of choline chloride superimposed with the N-H stretching of the amine groups of urea, which suggests the presence of hydrogen bonding between the components in the reline mixture (Figure S4a, dark green line). The C-H stretching mode of choline chloride is found in both constituent and reline spectra at about 2970 cm^{-1}. In the spectrum of reline, some peaks can be attributed to the urea, such as the C=O stretching absorption at 1650 cm^{-1}, and the strong sharp peak at 1007 cm^{-1} corresponding to the C-N bond vibrations. The strong peak at 760 cm^{-1} relative to the C–N stretching of the quaternary ammonium group in ChCl component was shifted to 710 cm^{-1} into reline.

6. Combined distribution functions (CDFs)

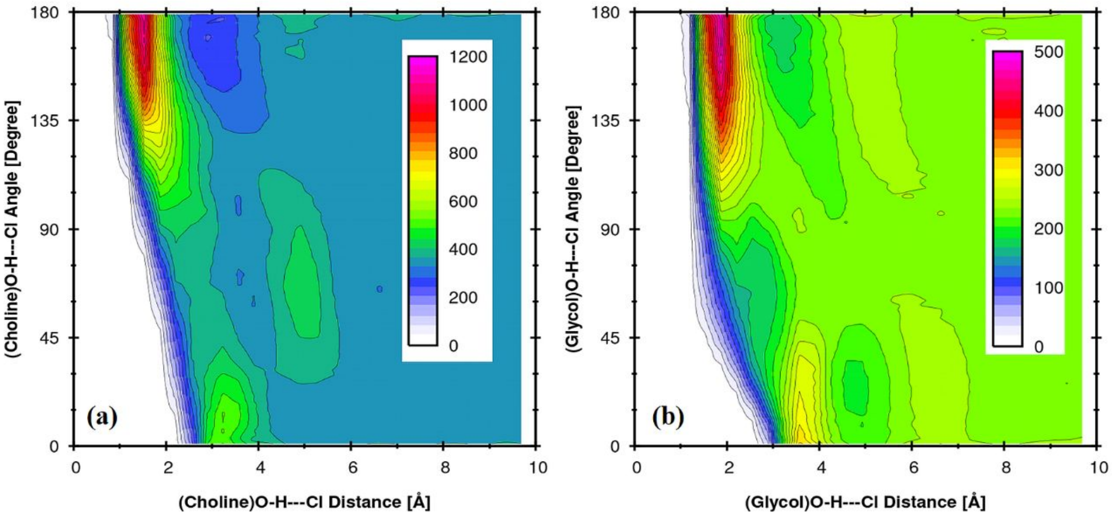

Figure S5. Combined distribution functions (CDFs, RDF vs ADF) hydrogen bonds in the Ethaline–GO system.(a) Choline O–H···Cl^-; (b) Glycol O–H···Cl^-

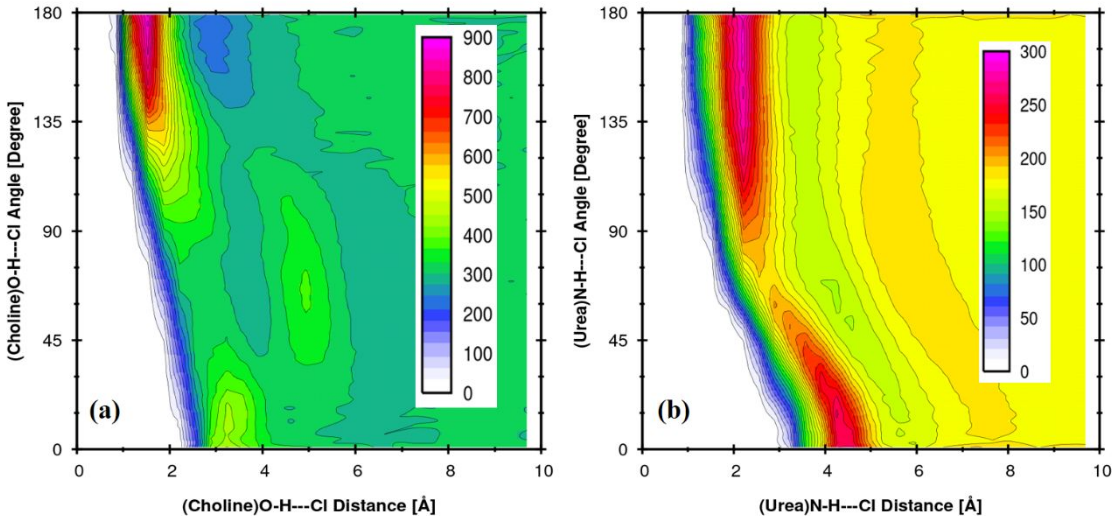

Figure S6. Combined distribution functions (CDFs, RDF vs ADF) for hydrogen bonds in the Reline-GO system. (a) Choline O–H···Cl⁻; (b) Urea N–H···Cl⁻

Figure S6 shows the angle–distance combined distribution functions for Cl⁻···H–X hydrogen bonds in the Reline–GO system. The O–H···Cl⁻ interaction involving choline (panel a) exhibits a relatively well-defined angular distribution, whereas the N–H···Cl⁻ interaction from urea (panel b) appears broader, indicating a greater variety of accessible geometries for the latter. This reflects the different conformational flexibility of the two donor species and the distinct nature of the hydrogen bonding involved.

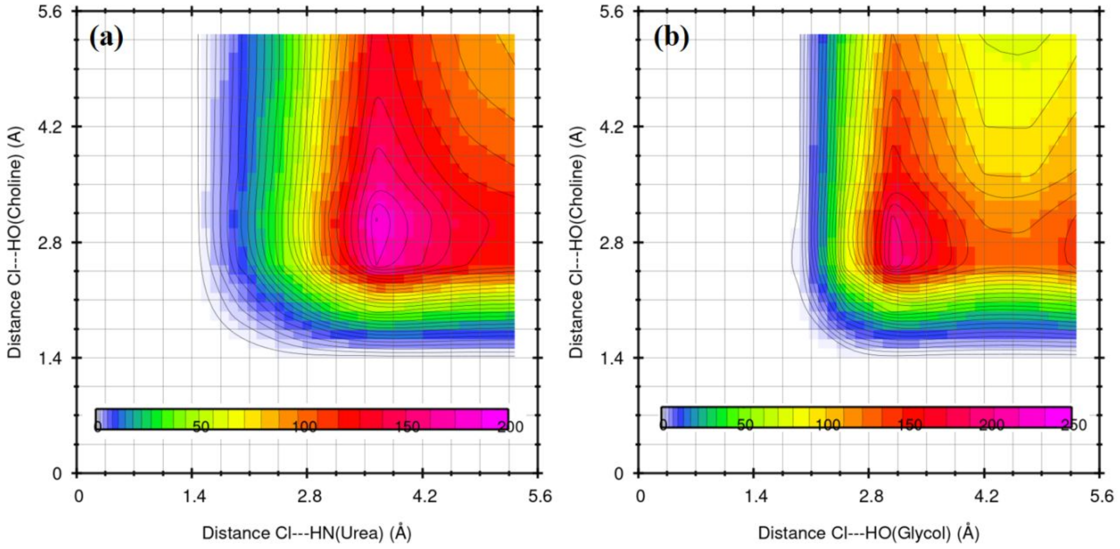

Figure S7. Combined distribution functions (RDF vs RDF) computed using Cl⁻ as the central reference, showing three-body spatial correlation between Cl⁻ and two HBD. (a) Distribution of Cl⁻–HO(choline) vs Cl⁻–HN(urea) distances in ethaline-GO. (b) Distribution of Cl⁻–HO(choline) vs Cl⁻–HO(glycol) distances in reline-GO

References

[1] G. Fioravanti, A. Galante, P. Fattibene, L. Torrieri Di Tullio, S. Colacicchi, G. De Thomasis, F. Perrozzi, N. De Berardinis, G. Profeta, L. Ottaviano, M. Alecci, Disentangling the intrinsic relaxivities of highly purified graphene oxide, Nanotechnology 35 (2024) 245101.

[2] N. Mehrabi, H.Q. Lin, N. Aich, Deep eutectic solvent functionalized graphene oxide nanofiltration membranes with superior water permeance and dye desalination performance, Chem Eng J 412 (2021) 128577.
